# Mechanisms of hysteresis in human brain networks during transitions of consciousness and unconsciousness: Theoretical principles and empirical evidence

**DOI:** 10.1371/journal.pcbi.1006424

**Published:** 2018-08-30

**Authors:** Hyoungkyu Kim, Joon-Young Moon, George A. Mashour, UnCheol Lee

**Affiliations:** 1 Department of Anesthesiology, University of Michigan Medical School, Ann Arbor, MI, United States of America; 2 Center for Consciousness Science, University of Michigan Medical School, Ann Arbor, MI, United States of America; Oxford University, UNITED KINGDOM

## Abstract

Hysteresis, the discrepancy in forward and reverse pathways of state transitions, is observed during changing levels of consciousness. Identifying the underlying mechanism of hysteresis phenomena in the brain will enhance the ability to understand, monitor, and control state transitions related to consciousness. We hypothesized that hysteresis in brain networks shares the same underlying mechanism of hysteresis as other biological and non-biological networks. In particular, we hypothesized that the principle of explosive synchronization, which can mediate abrupt state transitions, would be critical to explaining hysteresis in the brain during conscious state transitions. We analyzed high-density electroencephalogram (EEG) that was acquired in healthy human volunteers during conscious state transitions induced by the general anesthetics sevoflurane or ketamine. We developed a novel method to monitor the temporal evolution of EEG networks in a parameter space, which consists of the strength and topography of EEG-based networks. Furthermore, we studied conditions of explosive synchronization in anatomically informed human brain network models. We identified hysteresis in the trajectory of functional brain networks during state transitions. The model study and empirical data analysis explained various hysteresis phenomena during the loss and recovery of consciousness in a principled way: (1) more potent anesthetics induce a larger hysteresis; (2) a larger range of EEG frequencies facilitates transitions into unconsciousness and impedes the return of consciousness; (3) hysteresis of connectivity is larger than that of EEG power; and (4) the structure and strength of functional brain networks reconfigure differently during the loss vs. recovery of consciousness. We conclude that the hysteresis phenomena observed during the loss and recovery of consciousness are generic network features. Furthermore, the state transitions are grounded in the same principle as state transitions in complex non-biological networks, especially during perturbation. These findings suggest the possibility of predicting and modulating hysteresis of conscious state transitions in large-scale brain networks.

## Author summary

Hysteresis, characterized by distinct forward and reverse phase transitions, is ubiquitous in nature. For example, there are distinct temperatures for water freezing and ice melting. Similarly, it has been found that state transitions related to consciousness exhibit hysteresis. In particular, the concentration of general anesthetics required to achieve loss of consciousness is significantly higher than the concentration at which consciousness is regained. However, it is unknown whether this is trivially reducible to the pharmacology of these drugs or if it is something related to brain function itself. In this study, we took a novel, network-based approach and hypothesized that the hysteresis observed during anesthetic state transitions shares the same underlying mechanism as that observed in non-biological networks. Our computational modeling, analytic study, and high-density human EEG analysis suggest that various hysteresis phenomena during loss and recovery of consciousness can be explained in principled ways by generic network features. Identifying these network mechanisms of hysteresis in the brain also provides a unified framework for understanding the radically different conscious state transitions associated with sleep, anesthesia, and disorders of consciousness.

## Introduction

Hysteresis, the differential pathway of forward and reverse state transitions, is a universal phenomenon observed in nature and has been investigated in various fields such as physics, engineering, biology, and economics[1–12]. Hysteresis has also been observed during state transitions in the brain, such as sleep[6,7] and general anesthesia[8–12]. It has been found in both drosophila and murine models that the concentration of general anesthetic required to induce unconsciousness is higher than the concentration at which consciousness is regained. Thus, identification of the mechanism of hysteresis will be essential to the complete understanding of conscious state transitions, with clinical applications to fields such as anesthesiology and neurology.

Traditionally, it has been assumed that the induction of and emergence from general anesthesia are mirror images of one another. However, the asymmetric anesthetic concentrations associated with induction and emergence have long been recognized and explained by pharmacokinetics[13–17]. Recently, it has become clear that the neural circuits mediating loss of consciousness do not entirely overlap with the neural circuits mediating recovery of consciousness. Max Kelz proposed ‘neural inertia’ as a fundamental and biologically conserved principle by which neural circuits of the central nervous system resist behavioral state transitions, such as those between consciousness and unconsciousness[8]. Neural inertia suggests that hysteresis is not a pharmacokinetic attribute, but a fundamental neurobiological process that stabilizes states of consciousness and creates resistance to rapid and potentially catastrophic transitions[8]. Accumulating evidence indicates that the relevant sites mediating the induction of and emergence from anesthesia are distributed globally rather than localized in the brain[13]. If hysteresis is a large-scale network phenomenon in the brain then, like many other biological and physical systems, the state transitions may be governed by the same network mechanism, which is referred to as ‘explosive synchronization.’

Explosive synchronization is a discontinuous transition between incoherent and synchronized states of a network[18,19]. We previously demonstrated that healthy humans undergoing frequent loss and recovery of consciousness in a lightly anesthetized state demonstrate conditions of explosive synchronization in networks reconstructed from high-density EEG[20]. We also conducted a modeling study in human brain networks that suggests varying patterns of explosive synchronization as a mechanism of diverse state transition patterns[21]. Based on our work and that of others, we hypothesized that the hysteresis observed during the loss and recovery of consciousness might be mediated by patterns of explosive synchronization, as occurs in many other physical and biological systems.

To test our hypothesis, we analyzed high-density EEG data from 22 healthy human volunteers during consciousness, anesthetic-induced unconsciousness (ketamine, n = 15; sevoflurane, n = 7), and recovery as well as the transitions between them. To identify empirical evidence of hysteresis based on EEG, we examined the EEG networks during the loss and recovery of consciousness induced by anesthesia. Assuming that characteristic features of EEG networks (i.e., altered connectivity, high modularity, and reconfigured hub structures) observed during general anesthesia reflect brain states that influence behavioral responses[22], we examined the EEG network and its trajectories in a 2-dimensional parameter space consisting of topographic similarities and connection strengths (Table 1). We also developed a neuroanatomically informed brain network model to identify the onset points of the state transitions and the conditions for hysteresis during loss and recovery of consciousness. Finally, with empirical data and analytic study, we tested the control parameters that were identified in the model for their ability to modulate hysteresis patterns across conscious state transitions.

**Table 1:** Glossary.

Hysteresis	State transitions characterized by distinct forward and reverse pathways. In this study, hysteresis was defined as the disparity of forward and reverse trajectories related to topographic similarities and connection strengths of EEG networks during state transitions.
Explosive synchronization	The discontinuous transition between incoherent and synchronized states of a network. In this study, we implemented an adaptive feedback process to a coupled oscillatory brain network model that facilitates explosive synchronization.
Topographic similarity	Pearson correlation between the node degrees (i.e., the number of connections of nodes) of two EEG networks. In this study, we measured topographic similarity between the EEG networks during general anesthesia and that of the baseline. Topographic similarity reflects how far an EEG network under general anesthesia is away from that of the baseline.
Connection strength	The average node degree of a binary network. In this study, a binary EEG network was constructed with the phase locking between two EEG signals and a threshold estimating the functional similarity across brain regions. Together with topographic similarity, it defines the brain state at a time point, allowing the construction of a trajectory during state transitions.
Coupling strength (*S*)	A model parameter that modulates the covariation among anatomically linked nodes in the brain network. We controlled this parameter to simulate the reconfiguration of EEG networks by increasing and decreasing the level of network synchronization.
Power of adaptive feedback term (*Z*)	A model parameter that modulates the power of feedback processes in a network, which facilitates explosive synchronization. We controlled this parameter to modulate the critical points of state transitions between synchronized and incoherent states, which consequently alters the hysteresis size (e.g., larger *Z*, larger hysteresis). Analytically, we propose that this term in the model is equivalent to the Hill coefficient (reflecting the dose-response slope of anesthetics) in the dose-response equation.
Frequency range *(Δω)*	A model parameter that modulates the critical points of state transitions between synchronized and incoherent states, and alters the hysteresis size (e.g., larger *Δω*, larger hysteresis). In this study, we measured the variance of the peak frequencies of EEG signals, and tested its correlation with the recovery time (from the loss of consciousness to the recovery of consciousness) of each subject.

## Methods

### Ethics statement

This study was conducted at the University of Michigan Medical School and approved by the Institutional Board Review (HUM00061087); after careful discussion, written informed consent was obtained from all participants.

### Experimental procedures

We used high-density EEG data from two independent studies using ketamine and sevoflurane; detailed methodology can be found in the S1 Text and the previous studies by Vlisides et al[23] (ketamine) and Blain-Moraes et al[24] (sevoflurane). For this study, we selected four states (baseline, induction, unconscious, and emergence) from the state transitions of each data set. The hypotheses and related analyses of the current study were completely distinct from that of the original studies.

### Ketamine and sevoflurane dataset

Fifteen human volunteers with 128-channel EEG were studied in the ketamine experiment, which included a subanesthetic dose (0.5 mg/kg ketamine administered over 40 minutes) and general anesthesia (induced by a single bolus dose of 1.5 mg/kg). Seven volunteers with 64-channel EEG were studied in the sevoflurane experiment during anesthetic concentrations gradually increasing from 0.4% to 0.6% to 0.8% (the average concentration at which unconsciousness was induced in the original study) or beyond, then decreased from 0.8% to 0.6% in high-flow oxygen (8 L/min). The EEG in both experiments was recorded with eyes closed. The loss and recovery of consciousness were defined as the loss and recovery of response to the verbal command ‘squeeze your left [or right] hand twice,’ on a recording loop every 30 seconds, with right/left hand commands randomized.

The EEG includes four different levels of consciousness: baseline, induction, unconscious, and emergence. Since the lengths of induction and unconsciousness were variable across participants, we chose one EEG epoch for each state, which also enabled us to calculate the hysteresis areas in a parameter space. We determined that the selection of an EEG epoch for each state does not change the results qualitatively.

The states analyzed in this study are defined as:

1. Baseline: 5 minutes before anesthetic administration in both experiments,
2. Induction: first 10 minutes of the subanesthetic dose in the ketamine experiment, and 5 minutes before the loss of consciousness in the sevoflurane experiment,
3. Unconscious: 5 minutes during unconsciousness in both experiments,
4. Emergence: 5 minutes after the recovery of consciousness in both experiments.

The average reference was used for referencing and the windowed sinc-FIR filter (in the MATLAB toolbox from EEGLAB) was used to avoid a possible shifting of the signal phases in both analyses. We analyzed 2-minutes-long EEG epochs with 10-seconds-long moving windows for each state.

### Identifying network-level hysteresis during anesthetic state transitions

**Fig 1:**
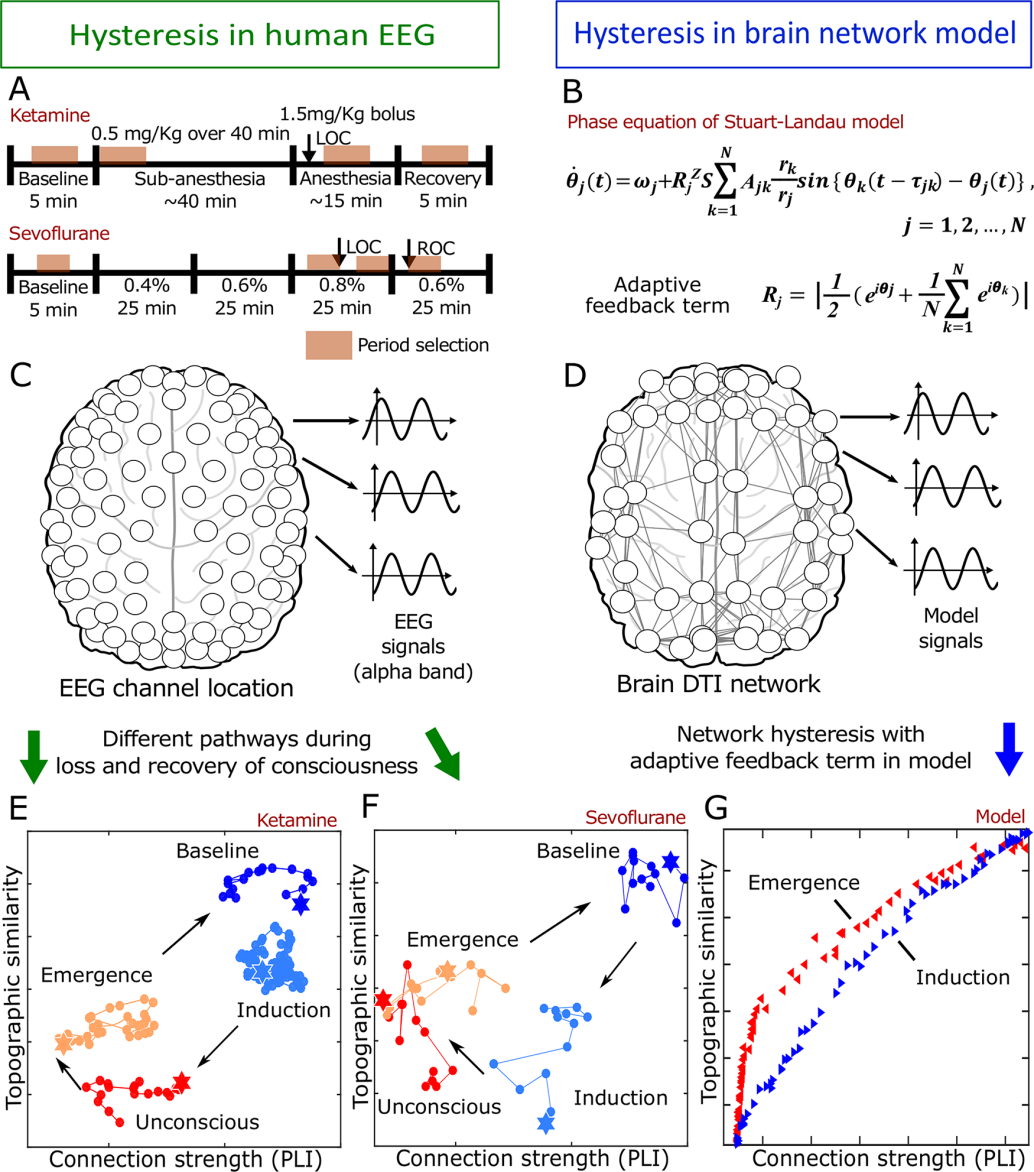
Schematic flow diagram for quantification of hysteresis in EEG and identification of the underlying mechanism of diverse hysteresis patterns during the loss and recovery of consciousness. The disparities of trajectories were acquired from two anesthesia experiments (sevoflurane and ketamine), and the strength and topological structure of the EEG network were used to track the state transition quantitatively. A large-scale human brain network implemented with an adaptive feedback process, one of the control parameters for hysteresis, was studied to identify the network mechanism of hysteresis.

The following procedure, illustrated in Fig 1, was implemented:

1. 2-minutes-long EEG epochs were selected during four different levels of consciousness: baseline, induction, unconscious, and emergence.
2. The functional connectivity was measured for various frequency bands of each state.
3. The connection strength and the topographic similarity of each state was measured with reference to the baseline.
4. The hysteresis areas in a 2-dimensional parameter space that consists of connection strength and topographic similarity were calculated for various frequency bands.
5. The hysteresis derived from the functional EEG network and a large-scale brain network model were compared with each other.

### Functional connectivity

Phase Lag Index (*PLI*), a measure of phase locking between two EEG signals, was used to define the functional connectivity in the EEG network[25]. We chose a Hilbert transform to extract the instantaneous phase of the electroencephalogram from each channel and calculate the phase difference *Δθ*_*ij*_(*t*) between channels *i* and *j*, where *Δθ*_*ij*_(*t*) = *θ*_*i*_(*t*) − *θ*_*j*_(*t*), *t = 1*,*2*,*…*,*n*, and *n* is the number of samples within one epoch. *PLI*_*ij*_ between two nodes *i* and *j* is then calculated using Eq (1):

$${PLI}_{ij}=|<sign\;({\Delta \theta }_{ij}(t))>|,\;0\leq {PLI}_{ij}\leq 1.$$ (1)

Here, the sign() function yields: 1 if *Δθ*_*ij*_(*t*) > 0; 0 if *Δθ*_*ij*_(*t*) = 0; and -1 if *Δθ*_*ij*_(*t*) < 0. The mean < > is taken over all *t = 1*,*2*,*…*,*n*. If the instantaneous phase of one signal is consistently ahead of the other signal, the phases are considered locked and *PLI*_*ij*_ ≈ 1. However, if the signals randomly alternate between a phase lead and a phase lag relationship, there is no phase locking and *PLI*_*ij*_ ≈ 0.

### Surrogate data

To control for spurious connectivity of EEG, 20 surrogate data sets were generated with a random shuffling method, in which a time point is randomly chosen in each EEG channel; the EEG epochs are then shuffled before and after the time point. The shuffled data have the same amplitude distribution and power spectrum of the original EEG but there are disruptions of the original connectivity between two EEG signals. The non-zero *PLI* from the shuffled data is regarded as spurious connectivity.

### Network construction

We expected that different EEG frequency bands and different states would have different levels of spurious connectivity[26]. Thus, after subtracting the median *PLI* of 20 surrogate data sets, if the remaining *PLI* was larger than 0.1 then the connectivity of two EEG signals was set as 1; otherwise, it was set as 0. The threshold (0.1) was chosen to avoid isolated nodes in the EEG network in the baseline states (S1 Fig). The basic EEG network properties were examined across states during the two anesthetic experiments. The node degree of an EEG channel was defined as the number of links in the network.

### Quantification of hysteresis in functional brain networks

**Fig 2:**
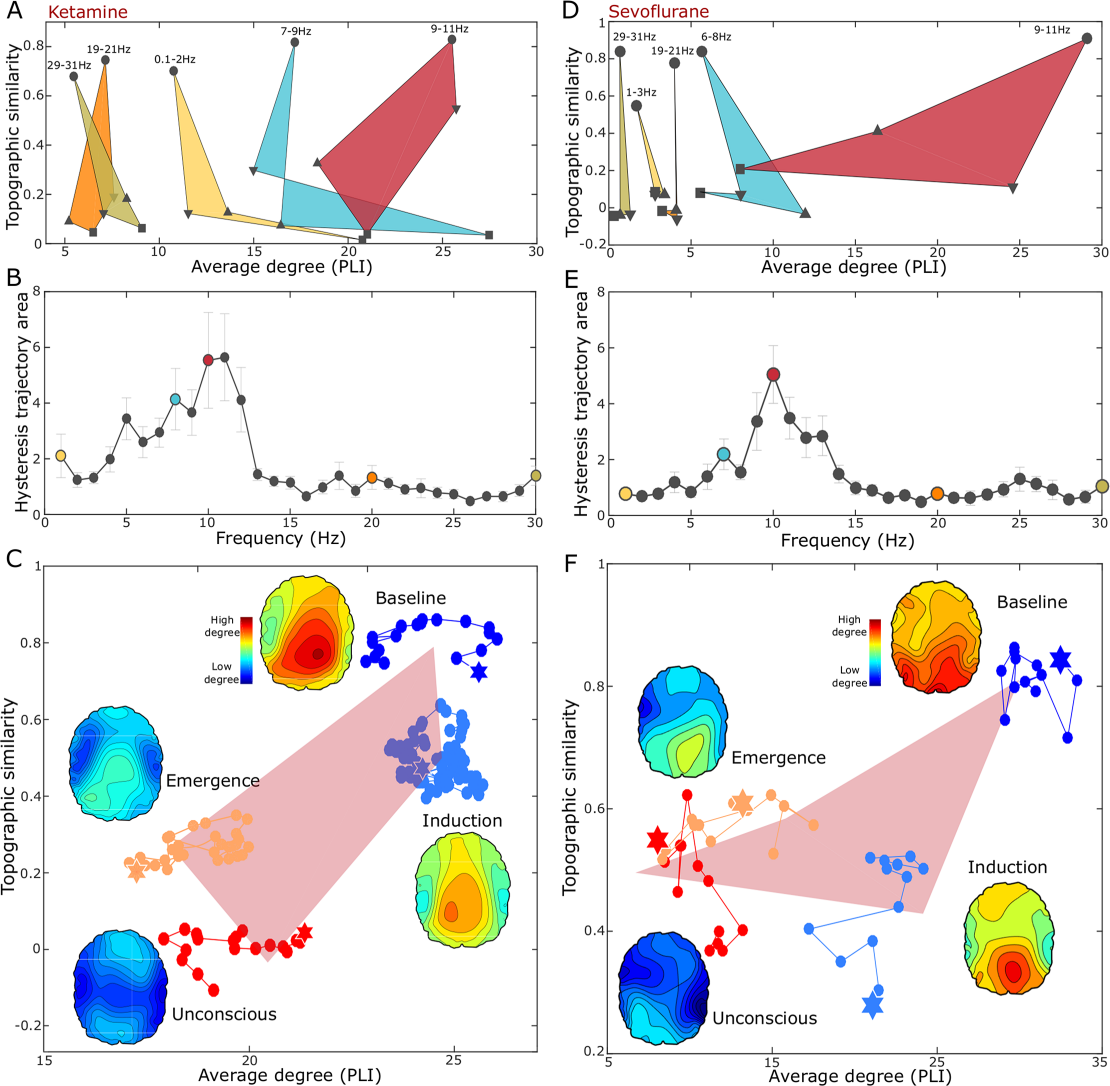
The hysteresis size of functional brain networks for different frequency bands. (A and D) Trajectories of topographic similarity and average node degree for five frequencies bands in each experiment. Each symbol in the trajectory indicates the four states, baseline (circle), induction (down-pointing triangle), unconscious (square), and emergence (up-pointing triangle) in both experiments, and the median of all individuals. The shaded area represents the hysteresis size of the functional brain network during state transitions. (B and E) The hysteresis sizes are calculated with 2 Hz frequency bins from 0.1 to 31Hz. The frequency range of 9-11Hz has the largest hysteresis size. The colors correspond to the trajectories shown in (A and D), respectively. The error bar indicates standard error. (C and F) The detailed trajectories of the frequency band (9-11Hz) within the four states are presented.

Temporal coordination of neural activity is a necessary condition for neural communication in the brain[27]. An EEG network constructed with phase lag index (phase synchronization) and its topography of node degrees may reflect a coarse-grained structure facilitating neural communication across brain regions. We previously demonstrated that the topography of EEG networks can differentiate various states of consciousness during general anesthesia[28]. Thus, in this study, we defined the brain state by the topography and the average node degree of an EEG network, which reflect the structure and strength of neural communication, respectively, across the brain regions associated with each EEG electrode. To quantify hysteresis, we constructed a two-dimensional parameter space that consists of the average node degree and topographic similarity of EEG networks, and then examined the trajectories of EEG networks in the parameter space (Fig 1). Topographic similarity measures how far the EEG networks under general anesthesia diverge from that of the baseline. Considering the large variability, we used a relatively long epoch length and small moving window size to represent the brain state. We segmented the 2-minutes-long EEG epochs with 10-seconds-long moving windows. The topographic similarity and the average node degree of EEG networks were calculated for each 10-seconds-long moving windows, and the median value of every epoch in the same state was used to represent a state in the parameter space. The hysteresis size for a given subject and anesthetic was calculated with the four median topographic similarities (representing four states) and average node degrees in the parameter space (Fig 2A and 2D).

Topographic similarity was defined by the Pearson correlation coefficient between the average node degrees of the baseline and the node degrees of each epoch from the four states (baseline, induction, unconscious, and emergence). Average node degree was defined by the average node degree of every node in each epoch.

$$\mathrm{Topographic}\;\mathrm{similarity}=Corr\;\left({\bar{B}}_{i,j},A_{i,j}\left(k\right)\right),\;i,j=1,\cdots ,N\;\mathrm{and}\;k=1,\cdots ,T.$$ (2)

$$Average\;node\;degrees=\frac{1}{N}\sum_{i}\left[A_{i,j}(k)\right],\;i,j=1,\cdots ,N.$$ (3)

where *A*_*i*,*j*_(*k*) is a binary connection matrix of each epoch and ${\bar{B}}_{i,j}$ is the averaged connection matrix over every epoch of the baseline. Corr (⋯) is the Pearson correlation coefficient function. i and j are the indices of every node (N) and k is the index of each epoch.

### Spectrogram analysis of experimental data

For all selected periods within each subject, spectral power was computed based on the short-time Fourier transform using the ‘spectrogram.m’ function in the MATLAB Signal Processing Toolbox (time window: 3s hamming window, overlap: 50%). The median absolute power (μV^2^/Hz) was then computed for each experimental period at selected frequency bands, for all channels.

### Statistical analysis

We performed one-way ANOVA (“anova1.m”, MATLAB toolbox) with Tukey-Kramer correction (“multcompare.m” with alpha = 0.05 and ctype = “tukey-kramer” in MATLAB) for the comparison of the hysteresis areas among various frequency bands. The statistical tests were carried out for each experiment separately. The adjusted P-values of 0.05 or lower (*P < 0.05, **P < 0.01, and ***P < 0.001) were considered to be statistically significant (S1 Table and S2 Table).

### Hysteresis in the human brain network model

The main goal of large-scale brain network modelling with simple oscillatory models is to identify general computational principles rather than to achieve biological realism. Our goal of the model study was to identify an underlying network mechanism of hysteresis phenomena and to test our hypothesis that the property of explosive synchronization identified in generic network models also holds for state transitions of the brain network during general anesthesia. Many recent studies have successfully applied Kuramoto/Stuart-Landau models to the brain in order to understand the organizational principles of multiscale brain function, surrogates of information flow, and complex dynamics at the whole brain network level[29–31]. Similarly, we believe that the application of simple oscillatory models to anatomically informed brain network structure can capture the essence of the hysteresis phenomena.

To model the hysteresis phenomena that are empirically observed during anesthetic state transitions, we used a large-scale brain network model that implements a general coupled oscillator model on the scaffold of an anatomically informed human brain network structure. The human brain network consists of 78 parcels of the cerebral cortex constructed from diffusion tensor imaging (DTI) of 80 young adults[32]. Previous studies with a Kuramoto model demonstrated that the fraction of the nodes adaptively controlled by local order parameters (average phase synchronization of a node with its linked nodes) modulates the hysteresis during state transitions between incoherent and synchronized states[19,33,34]. The result was robust for various network configurations. Here, we extended the Kuramoto model with a modified Stuart-Landau model that includes an adaptive feedback term, i.e., a recursive interaction process between a node and the other nodes. The modified Stuart-Landau model shows how an adaptive feedback term modulates the hysteresis in both the phase and amplitude dynamics.

$${\dot{r}}_{j}(t)=\{\lambda_{j}-{\left|r_{j}(t)\right|}^{2}\}r_{j}(t)+S\sum_{k=1}^{N}A_{jk}r_{k}\;cos\;\left(\theta_{k}(t-\tau_{jk})-\theta_{j}(t)\right),$$ (4)

$${\dot{\theta }}_{j}(t)=\omega_{j}+{R_{j}}^{Z}S\sum_{k=1}^{N}A_{jk}\frac{r_{k}}{r_{j}}\sin\;\left(\theta_{k}(t-\tau_{jk})-\theta_{j}(t)\right)\;,j=1,2,\ldots ,N.$$ (5)

Here *r*_*j*_*(t)* is the amplitude of oscillator *j* at time *t*. *λ*_*j*_ is a parameter governing the amplitude of each oscillator. *S* is the coupling strength between oscillators and A_*jk*_ denotes the anatomical connections between oscillator *j* and *k*, yielding 1 if a connection exists and 0 otherwise. *τ*_*jk*_ is the time delay between node *j* and *k*. *θ*_*j*_*(t)* is the phase of oscillator *j* at time *t*. *ω*_*j*_ is the intrinsic frequency of oscillator *j*. We modified the original Stuart-Landau model by adding the *R*_*j*_^*Z*^ term. We define *R*_*j*_ as synchrony of node *j*: $R_{j}\equiv |1/2(e^{i\theta_{j}}+1/N\;\sum_{k=1}^{N}e^{i\theta_{k}})|$, to measure the extent to which the node *j* is synchronized with the other nodes. In the setting of a heterogeneous network like the brain, *R*_*j*_ avoids a bias of the local order parameter. For instance, the local order parameter of a node whose node degree is 1 is determined by the phase synchronization of only the linked node and its initial frequency, randomly arranged. *R*_*j*_ is defined to be confined between 0 and 1, where 0 signifies complete incoherence and 1 means complete synchronization. *Z* is a scale term for the feedback process between a node *j* and its linked nodes. The adaptive feedback process incorporates the memory of a given state of synchronization into the dynamics, simultaneously enhancing heterogeneity in the phase and amplitude dynamics of the brain network. Notably, the adaptive feedback term was multiplied by the coupling strength of the phase equation, not by the amplitude equation. However, the phase and amplitude dynamics mutually interact with each other in Eqs (4) and (5). Direct implementation of a feedback term in the amplitude Eq (4) causes a divergence.

We analytically and computationally showed that hysteresis naturally occurs during state transitions between incoherent and synchronized states with mean field approximation[19,31,35], and identified the control parameters that modulate the hysteresis. In this work, the degree of synchrony will be measured by global order parameter $R\equiv |1/N\;\sum_{k=1}^{N}e^{i\theta_{k}}|$. The analytic derivation is shown in the S1 Text. We extended the previous studies in several ways. First, with the modified Stuart-Landau model, we can study the hysteresis in phase dynamics and amplitude dynamics independently, which enabled us to interpret the hysteresis in the phase-based connectivity and EEG power during state transitions independently. Second, the newly defined node synchrony considers heterogeneous local network connectivity and *Z* in the adaptive feedback terms as associated with the steepness of dose-response slope of anesthetics. Third, from the model studies, we can infer the range of frequency *Δω* as another control parameter to modulate hysteresis during the state transition. Fourth, we analytically derived the specific feedback term, *R*_*j*_^*Z*^*S*, from a pharmacokinetic equation (i.e., Hill coefficient equation), suggesting the role of the feedback term as the anesthetic effect on brain network synchronization (the analytic derivation is in S1 Text).

### Simulation procedures

All parameters for the models were set to simulate alpha oscillations in the brain, because the hysteresis areas were highest in the alpha peak (9-11Hz) in the experimental results. The natural frequencies of the oscillators in our simulation were given as a Gaussian distribution around 10 Hz with standard deviation of 1 Hz. Time delay was given proportional to the physical distances between edges with a propagation speed of 8.6m/s[36]. The coupling strength between the oscillators was continuously increased from 0 to 50 and decreased from 50 to 0 for continuous change between a fully synchronized and fully unsynchronized network. The amplitude parameter λ_j_ was given identically for all oscillators with a value of 1. For all simulations, we also added a Gaussian white noise variable ξ_j_(t) with a mean and standard deviation of 2 Hz. We changed the power of the adaptive feedback term Z from 2 to 8, and the distribution range of natural frequencies Δω from 0.1 to 4, in order to investigate the relationship between the parameters and hysteresis size. In each parameter set, 200 configurations were simulated and the results were averaged over all configurations.

## Results

### Quantification of hysteresis phenomena in EEG networks

To find empirical evidence for hysteresis phenomena at the network level, we measured *average node degree* and *topographic similarity of node degree*. These variables represent the connection strength and structure of the functional network, respectively, and have been demonstrated to help differentiate levels of consciousness in the context of anesthetic state transitions[28]. The two measures were used to construct a trajectory of functional brain networks during the loss and recovery of consciousness.

For the trajectory, we constructed the functional networks with 2-minutes-long EEG epochs and 10-seconds-long moving windows. Phase Lag Index (PLI) [25], a simple phase locking measure between two EEG signals, was used to define the edges in the functional network. For each epoch, we computed average node degree and topographic similarity. Topographic similarity measures the correlation between the node degrees of the baseline state and the node degrees of each epoch for every node in experiment 1 (ketamine) and experiment 2 (sevoflurane). The change of topographic similarity indicates how the connectivity structure changes during general anesthesia compared to the baseline state.

Fig 2A and 2D show the trajectories of the EEG network for different frequency bands during the state transitions in both experiments (from baseline (circle) through induction (down-pointing triangle), unconscious (square), and emergence (up-pointing triangle)). The point representing each state is the median value of all individual subjects. The states of individual subjects were calculated with an average of every epoch. Each EEG frequency band shows a distinctive hysteresis pattern. Fig 2B and 2E present the hysteresis sizes of different frequency bands (2Hz frequency bins for 0.1-31Hz frequency range). The hysteresis size was defined by the area encompassed by the forward and reverse pathways. The result shows that the alpha band (9-11Hz) has the largest hysteresis compared to the other bands (about 5-fold larger than the smallest hysteresis). Fig 2C and 2F shows the trajectory of the alpha band. Each marker represents the average node degree and average topographic similarity over the 15 (ketamine) and 7 (sevoflurane) subjects for each epoch. Filled stars represent the first epoch within each state. The brain topographic map for each state represents the node degree pattern averaged over all subjects. Notably, in the two experiments, the trajectories turn clock-wise, which implies that the topographic similarities during emergence are always higher than during the induction path at the same connection strength. In other words, the connection structure responds more sensitively to the induction and emergence than the connection strength. There is evidence that general anesthetics might disproportionately affect hub structures, which occupy a relatively small portion of the brain network but play a significant role in information transmission. Hub disruptions (leading to faster or slower dynamics) change functional connection structure at an earlier stage of anesthesia, while the average connection strength is still intact. A significant change in average connection strength follows later with overall damage of functional networks. Such differential responses to a perturbation of connection structure and strength is a generic feature for a heterogeneous network that is perturbed.

### Comparison between the simulation and empirical data

**Fig 3:**
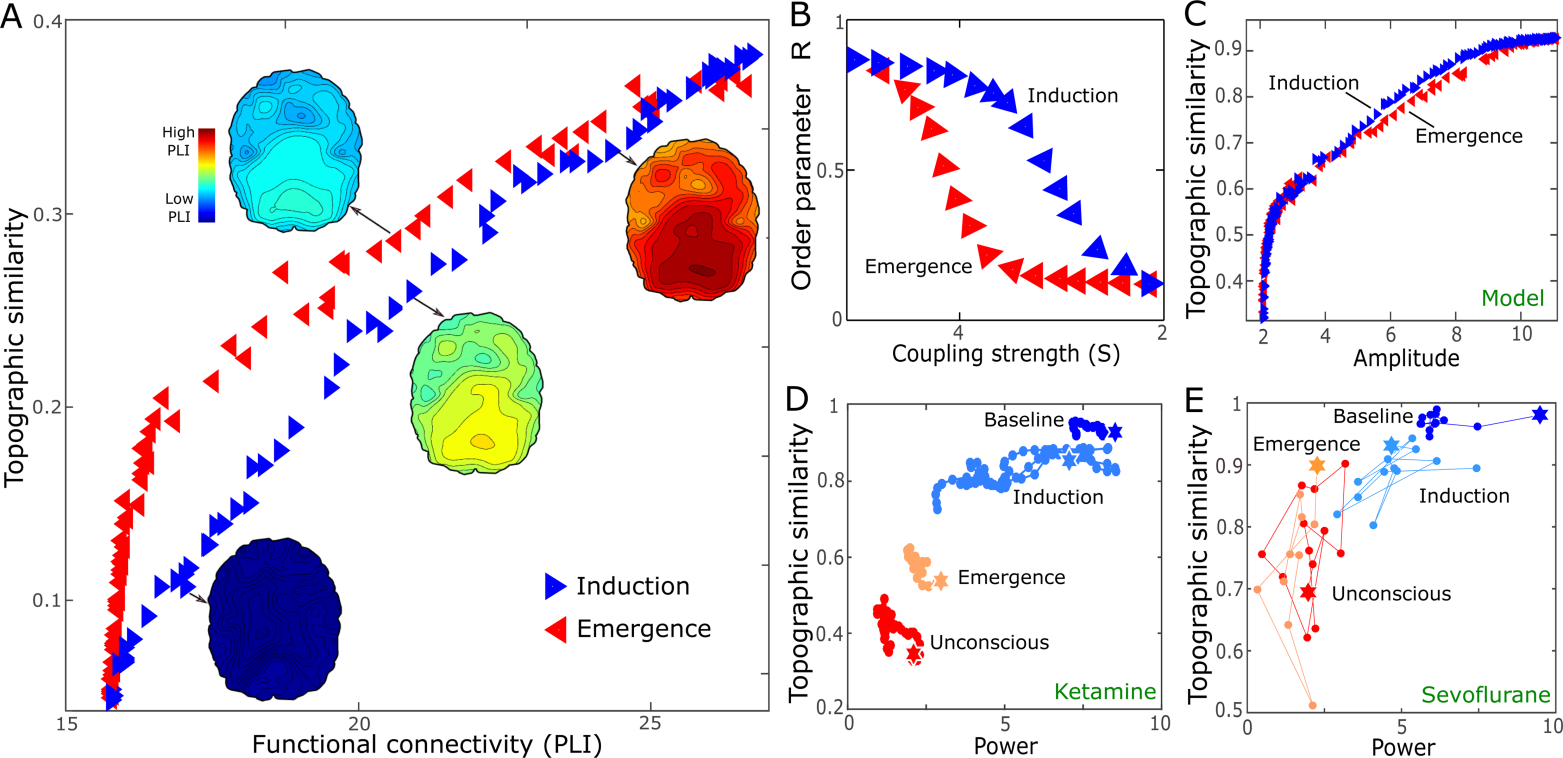
Hysteresis in the brain network model. (A) The trajectory in the 2-dimensional space also demonstrates a significantly large hysteresis. The topographic similarity during the increase of the coupling strength *S* (red left-pointing triangle) is always higher than that during the decrease of the coupling strength (blue right-pointing triangle). (B) The synchronization level (the order parameter R) showed a hysteresis as the coupling strength S varies. The trajectory in 2-dimensional space constructed with the power does not demonstrate a significant hysteresis in either the model data (C) or the empirical data (D and E). This result implies that hysteresis is mainly due to the global connections rather than local variables such as power. The model parameter for (B) and (C) is *Δω* = 2 and *Z* = 4.

In order to compare the simulation and empirical data, we assumed brain networks with nodes that are synchronized correspond to conscious states and networks with incoherent nodes correspond to the unconscious state induced by general anesthetics. The empirical data support this assumption; deep anesthesia reduces the global synchronization level of the brain network. In Fig 3A and 3B, we simulated the typical state transition pattern (i.e., the clock-wise turn of trajectories) observed in the empirical data in Fig 2C and 2F. The topographic similarity during the emergence period is always higher than the induction period for the same average node degree. When changing the coupling strength *S* in the brain network model, the order parameter *R* shows a hysteresis in both forward and reverse pathways (Fig 3B). When *S* increases from 0, the transition to a synchronous state occurs at a critical coupling strength *S*_*inc*_. However, when the coupling strength decreases, the transition to the incoherent state begins at a different critical coupling strength *S*_*dec*_. Analytic and simulation studies show that the transition points of *S*_*inc*_ and *S*_*dec*_ are not equal, indeed, *S*_*inc*_ < *S*_*dec*_, showing that, at each value of coupling strength *S*, the state of the system is not defined uniquely. In other words, if the synchronization level of a network is changed with an adaptive feedback process, then it naturally produces a path-dependence during state transition. The details of the analytic derivation for the different transition points, *S*_*inc*_ and *S*_*dec*,_ will be explained in the last section. Consequently, when we convert the typical path-dependence in the order parameter *R* (Fig 3B) into the trajectories of the topographic similarity and the connection strength (PLI), it manifests as a clock-wise turn (Fig 3A).

Furthermore, hysteresis is observed in the brain network connectivity but not the power. Here, we defined *average power* as the average over the powers of all nodes and the *topographic similarity of power* as the correlation of the power topographies between baseline and all epochs. Fig 3C presents the trajectory in the 2-dimensional space of the averaged power and the topographic similarity of power. For both the model data (Fig 3C) and empirical data (Fig 3D and 3E), no significant hysteresis was found. This result suggests that the hysteresis in the human brain is mainly due to global interactions across brain regions, rather than activities within brain regions.

### Two control parameters that modulate the hysteresis pattern

**Fig 4:**
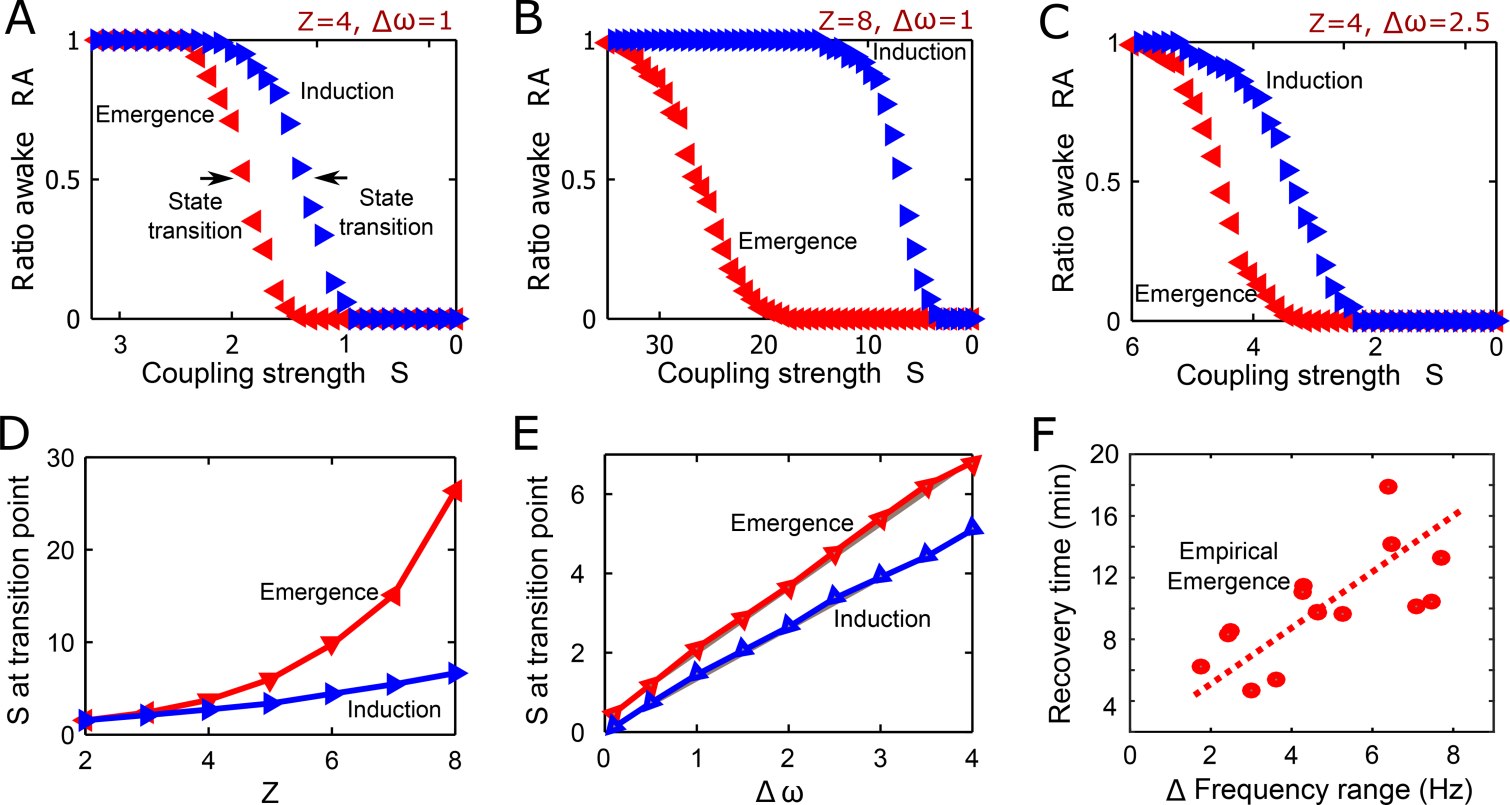
The modulation of hysteresis size with change of adaptive feedback process and frequency range (*Δω*). (A) The power coefficient *Z* = 4 and *Δω* = 1. The arrows indicate the onsets of state transition between incoherent and synchronized states that we defined. Blue triangles (blue left-pointing triangle) show the path of decreasing coupling strength *S* (induction), whereas red triangles (red right-pointing triangle) show the path of increasing coupling strength *S* (emergence). (B) *Z* = 8 and *Δω* = 1, and (C) *Z* = 4, *Δω* = 2.5. (D) The stronger adaptive feedback process with larger *Z* produces larger hysteresis, with different onset coupling strengths in the human brain network. (E) The critical coupling strengths of losing and recovering synchronization level linearly increases with *Δω*. (F) The recovery time from unconsciousness and the variance of the peak frequencies of human EEG shows a significant correlation (0.73, p<0.01, Spearman coefficient).

According to the model, we tested two control parameters, (1) the strength of adaptive feedback process, i.e., the power coefficient *Z* in the model, and (2) the variance of peak frequencies among nodes *Δω* in the brain network. We studied how the two control parameters modulate the onset points of state transitions between incoherent and synchronized states. As expected, Fig 4A and 4B show that a larger *Z* produces larger hysteresis (*Z* = 4 and 8 for Fig 4A and 4B, respectively). The *Z* value reflects the strength of an adaptive and recursive feedback process. The locally different feedback process with *R*_*j*_^*Z*^ promotes the heterogeneity of the network dynamics and modulates the onsets of state transitions. To determine the onset of the state transition, we defined the synchronized state as *R =* 0.5 and the critical coupling strength *S* as *R* = 0.5. The simulation was repeated 200 times with random initial frequencies and the results were robust with any other thresholds between 0.1 and 0.9. The mean of the frequencies was set as 10Hz with variance between 0.1 and 4 Hz to emulate the EEG of the alpha band. Fig 4D demonstrates the critical coupling strengths of both pathways with respect to *Z*. The critical coupling strengths increase along with *Z*. Notably, the critical coupling strength more steeply increases during emergence compared to induction. This implies that a stronger adaptive feedback process delays the onset of emergence from the incoherent state and has less influence on the onset of induction.

Second, the variance of frequency *Δω* is another control parameter that is measurable from EEG. The variance of initial frequency *Δω* in the model positively correlates with the critical coupling strengths in both forward and reverse pathways. Fig 4A and 4C present small and large variance of frequencies, *Δω* = 1 and 2.5, respectively. In the comparison, the larger *Δω* has larger critical coupling strengths than the smaller *Δω*. Fig 4E shows that the critical coupling strengths linearly increase with *Δω*. According to the relationship, if we assume that the coupling strength of the system is increased and decreased continuously between 0 and 6, we can predict that a network with a larger *Δω* would transition more easily at induction but resist emergence. Conversely, a network with a smaller *Δω* may have resistance to induction but be more permissive for emergence. For instance, the network of *Δω = 4* easily reaches its critical coupling strength during the induction (from 6 to 5 in Fig 4E), whereas in the network of *Δω = 1* it is relatively hard to reach the critical coupling strength (from 6 to 1.5). Conversely, it is difficult for the network of *Δω = 4* to reach its critical coupling strength during the emergence (from 0 to 6 in Fig 4E), but relatively easy for *Δω = 1* (from 0 to 2). To empirically test this model prediction, we investigated the correlation between the *Δω* of EEG during anesthesia and the recovery time, that is, the duration between the loss and recovery of consciousness. The test was carried out only with the data from the ketamine experiment, in which we could determine the recovery time precisely after administering the anesthetic, because ketamine was given as a single bolus as opposed to the downward titration of sevoflurane. Fig 4F demonstrates significant correlation between the recovery time and the *Δω* of the EEG (0.73, p<0.01, Spearman coefficient). The clear relationship between recovery time and the frequency range of EEG matches the model prediction.

### The mechanism of hysteresis in functional brain networks

A strong pharmacological perturbation such as general anesthesia induces a significant decrease of long-range network synchronization, which has been proposed as a neural mechanism of the loss of consciousness; the converse situation for the recovery of consciousness has been proposed [37–39]. The analytic derivation with mean-field approximation explains why the critical coupling strengths in both pathways are different as the synchronization level varies [33]. In the model, the adaptive feedback process plays a key role in the hysteresis. As *S* decreases, the inherent frequencies at which the oscillators join a synchronized cluster is smaller than certain value: *ω*_*j*_ *< Ω*_*dec*_ where *Ω*_*dec*_ *≈ S* ${\tilde{R}}^{2}k_{j}$. By contrast, as S increases, the permissive condition to join a synchronized cluster is *ω*_*j*_ *< Ω*_*inc*_ where *Ω*_*inc*_ *≈ S* $\tilde{R}k_{j}$. Here, $\tilde{R}$ is an arbitrary normalized synchronization measure (0 to 1) that is used as the adaptive feedback term in the model and *k*_*j*_ is the degree of node *j*. In the solutions, if the $\tilde{R}$ is near 1, the difference between *Ω*_*dec*_ and *Ω*_*inc*_ becomes negligible, but if *R* is near 0, the difference between *Ω*_*dec*_ and *Ω*_*inc*_ becomes larger, and *Ω*_*dec*_*< Ω*_*inc*_. Therefore, when the system transitions from an incoherent to a synchronized state (from $\tilde{R}\approx 0$ to $\tilde{R}\approx 1$), the possible number of oscillators to join a synchronized cluster is smaller than in the case of the opposite transition (from $\tilde{R}\approx 1$ to $\tilde{R}\approx 0$). In other words, as a network transitions from an incoherent to synchronized state, it is harder to reach the synchronized state (defined by the threshold), but in the opposite path it is easier to get to the desynchronized state. Such asymmetry in the possible number of nodes that can join a synchronization cluster along forward and reverse pathways is the key network mechanism of the hysteresis. As a result, hysteresis naturally appears when a network contains a feedback process.

## Discussion

Hysteresis is a universal phenomenon observed in many biological and physical systems[1–12]. In the brain, the hysteresis phenomenon is observed in various state transitions that can be spontaneous (sleep-wake cycle) or pharmacologically induced (general anesthesia) [6–12]. Hysteresis has been reported at various scales of observation in the brain [8–12,40,41]. At the molecular level, models of general anesthesia suggest that hysteresis may be caused by heterogeneous site effects resulting in pharmacokinetic/dynamic delays[13–17]. At the neuronal circuit level, Steyn-Ross el al. proposed that hysteresis naturally occurs as a first-order phase transition (i.e., discontinuous state transition) in the population average membrane voltage of cortical neurons[40]. A recent study in rodents reported distinctive sequences of thalamic and medial prefrontal cortex activity at loss and recovery of consciousness[42]. At the behavioral level, it has been demonstrated that different anesthetic concentrations are associated with loss and recovery of consciousness in diverse species such as mouse and fruit fly. As a result of this and other work, Kelz suggested a fundamental neurobiological process referred to as ‘neural inertia,’ which helps to maintain aroused and anesthetized states and creates resistance to state transitions[8]. Proekt and Hudson, using a mathematical model, tested the hypothesis that neural inertia is a consequence of the stochastic switching between the waking and anesthetized state. They showed that properties of a bistable system can account for phenomena related to neural inertia, supporting the hypothesis that emergence from anesthesia is independent of pharmacokinetic factors[43,44].

In this study, we hypothesized that the hysteresis observed during state transitions induced by general anesthesia is a generic network feature. Furthermore, we hypothesized that the state transition between consciousness and unconsciousness in the human brain may be governed by the same principle that is operational in non-biological complex networks responding to perturbations. Even though the molecular mechanisms of ketamine and sevoflurane are significantly different, we found that the trajectories of both state transitions were similar to each other, showing a clock-wise turn in the 2-dimensional parameter space composed of average node degree and topographic similarity. The consistent trajectory pattern of functional brain networks suggests a common mechanism of state transition during the loss and recovery of consciousness. We discovered in the model study that the larger topographic similarity of functional brain networks during the emergence process compared to that of the induction process is a spontaneous phenomenon, while the synchronization level decreases and increases in a network with an implemented feedback process.

In the EEG analysis, we also found that the lower frequency bands (<12Hz) showed significantly larger hysteresis, whereas the higher frequency bands (12- 30Hz) have a smaller hysteresis that approaches randomness. Notably, the alpha frequency band (8-12Hz) showed the largest hysteresis (about 5-fold larger than the smallest hysteresis in the high frequency band). The role of the alpha band during anesthetic state transitions is still unclear but the anteriorization process during propofol anesthesia and surgical levels of sevoflurane anesthesia has been well described[45]. Furthermore, a prominent response of the diverse functional connectivity measures in the alpha band has been reported during the loss of consciousness[24,46–49].

The large hysteresis of the alpha band can be explained by the fact that alpha oscillations are associated with global brain connectivity. For example, Zhang et al. demonstrated that the travelling waves of the alpha band propagate across brain regions[50]. The direction and the speed of traveling waves are determined by a spatial gradient of local frequencies and connections that also correlate with the performance of a memory task. Independently, we identified a general relationship between brain network structure and direction of connectivity[30,31]. Among the frequency bands, only the alpha bandwidth of the human and monkey brains matched the model predictions. These two studies demonstrate the association of alpha oscillations with global connectivity in the brain. Although anesthetics can affect all EEG frequencies, alterations of other frequency bands are relatively small because their original connectivity is significantly lower than the alpha band[23,48]. Since we defined hysteresis based on an EEG network derived with a particular kind of functional connectivity measure, the alpha band predictably shows the largest alteration of EEG network, which would allow the largest hysteresis to be observed relative to other frequency bands.

Interestingly, the hysteresis was observed only in the functional connectivity, not in the power of EEG. The empirical data in this study thus suggest that hysteresis is a function of global interactions, not regional brain activities. These empirical findings motivated us to develop the phase-based human brain network model and to investigate the generic features of hysteresis phenomena.

### Mechanism of hysteresis in human brain networks

In the field of statistical physics, especially in condensed matter physics, the hysteresis phenomenon during state transitions has been studied extensively[1,2]. Recently, studies of hysteresis have extended to network science, including the underlying network mechanism of explosive synchronization[18,19,35,51]. Filatrella et al. and Zhang et al. suggested that the suppression of giant synchronization cluster formation in a network is a general mechanism of explosive synchronization[33,35]. Under the conditions of such a synchronization suppression process, a network will exhibit explosive synchronization, i.e., a discontinuous state transition between incoherent and synchronized states, during which a hysteresis can occur. Incorporating previous studies, we developed a global brain network model of hysteresis. Our model generalizes the previous phase dynamics models[33,35], including the amplitude dynamics as well as time delays between oscillators. The model of amplitude dynamics was able to recapitulate the empirical finding. In particular, we empirically demonstrated that there was no hysteresis in the power of EEG, lending credibility to the model. The adaptive feedback term in the model plays an essential role in producing asymmetric suppression during destruction and reformation of giant clusters during the synchronization process. The feedback primarily acts on the phase synchronization rather than the amplitude of the oscillators. Our analytic solution for the model quantitatively explains the asymmetric pathways of a hysteretic state transition. When a network changes its overall level of synchronization, the threshold required to join a synchronized cluster is different in the forward and reverse pathways (*Ω*_*dec*_ *≈ S* *Ω*_*dec*_ *≈ S* ${\tilde{R}}^{2}k_{j}$ for decreasing synchronization; *Ω*_*inc*_ *≈ S* *Ω*_*dec*_ *≈ S* $\tilde{R}k_{j}$ for increasing synchronization). For instance, when the network transitions from an incoherent to synchronized state (from $\tilde{R}\approx 0$ to $\tilde{R}\approx 1$), the possible number of oscillators to join a synchronized cluster is smaller than that of the opposite transition (from $\tilde{R}\approx 1$ to $\tilde{R}\approx 0$). This discrepancy is greatly amplified at small $\tilde{R}$ (<<1) with *Ω*_*dec*_ (${\tilde{R}}^{2}$) << *Ω*_*inc*_ ($\tilde{R}$). Considering the fact that a consistent effect of general anesthetics is to decrease the temporal coordination in large-scale brain networks, we expect that the network mechanism of hysteresis may be more influential during the state transition around a lower level of consciousness.

### Brain synchronization and behavioral response

**Fig 5:**
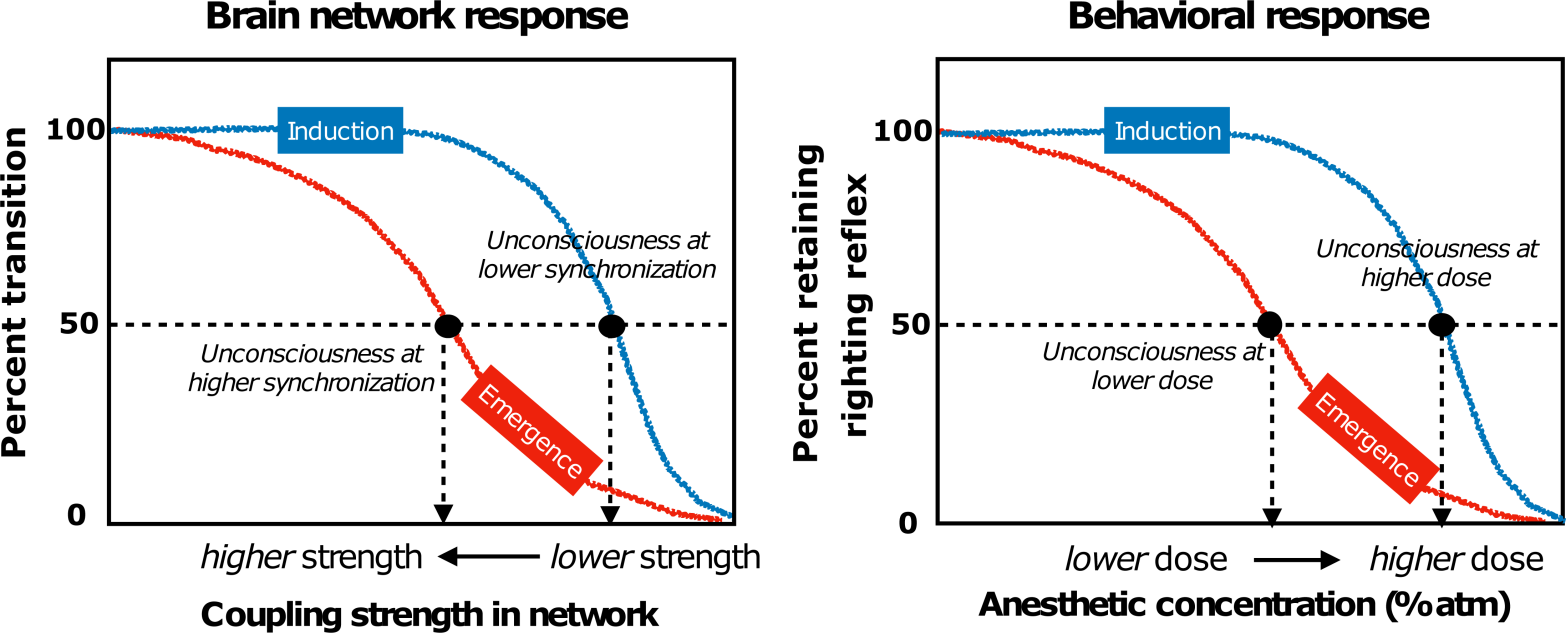
Hysteresis patterns in brain network responses and behavioral responses (as identified in rodents) during general anesthesia. The brain network state transition is defined by the percent crossing the synchronization threshold and the behavioral response is defined by the percent retaining righting reflex. The analytic derivation explains that the asymmetric pathways result from distinct criteria to join a synchronized cluster in a network.

The brain requires a higher anesthetic concentration to lose consciousness than to maintain unconsciousness. That is, despite the same behavioral response ratio, there can be different anesthetic concentrations depending on whether the pathway is moving in the forward or reverse direction[8]. In the same way, the brain network requires a higher coupling strength to restore synchronization (i.e., crossing over the threshold of state transition, *R* = 0.5) than to lose synchronization. Applying this to empirical observations of anesthetic state transitions, a lower anesthetic concentration during emergence would be more potent because the network now has higher thresholds to cross in order to resynchronize. However, the state transitions empirically observed during the loss and recovery of consciousness are not linear and monotonic but rather nonlinear and complex; here we simplified the problem as much as possible to identify a general network feature in the empirical data. In Fig 5, we present a conceptual schema to correlate the hysteresis pattern of the brain network with the hysteresis pattern of the behavioral response.

### Control parameters that modulate the hysteresis pattern

Another benefit of our model study is that it identified the two control parameters for modulating the hysteresis size. The *Z* value in the model reflects the strength of the adaptive feedback process in the brain, which relates to the dose-response slope of anesthetics. The simulation result demonstrated that a larger adaptive feedback process gives rise to a larger hysteresis. The dose-response curve for each anesthetic has a unique slope (*H*; Hill coefficient in Hill equation determines the dose-response curve). For instance, the *H* of halothane is 10–30 and that of isoflurane is 3–4. The hysteresis of halothane is larger than isoflurane[8]. The Hill equation determines the degree of cooperativity of the ligand binding to the enzyme or receptor. The Hill coefficient *H* quantifies the degree of interaction between ligand binding sites and measures the sensitivity of the response curve[52,53]. Analytically, the power coefficient *Z* of *R*^*Z*^ is equivalent to the Hill coefficient *H* in the dose-response curve for the anesthetic (S1 Text). However, to biologically link the adaptive feedback term Z in our model and the Hill coefficient *H* in the dose response curve will require further investigation.

Our model also suggests the variance of frequencies as another control parameter that modulates hysteresis in functional brain networks. We found a strong correlation between the variance of EEG peak frequencies during anesthesia and the onset of recovering consciousness in the empirical analysis of the ketamine experiment. Larger variance of the peak frequencies of channels was associated with a more delayed onset of recovering consciousness. This is consistent with our model prediction, i.e., larger variance of frequencies lead to larger hysteresis with relatively delayed onset of a state transition from the incoherent to synchronized state. Our model prediction also holds for the opposite pathway. Chennu et al. demonstrated that subjects who have lower EEG coherence (weaker phase-based connectivity in the alpha band) at baseline were more likely to become unresponsive during sedation[49]. The model with a larger variance of frequencies demonstrated that the brain network is less synchronized at a higher coupling strength, i.e., a relatively small perturbation of coupling strength induces a transition into an incoherent state. This may explain why individuals with lower functional brain connectivity, which can be caused by a large variance of EEG frequencies, lose consciousness more easily.

### Relationship between the steepness of dose-response slope of anesthetics and adaptive feedback term in the model

Conventionally, the dose-response slope of the anesthetic is described with the Hill coefficient of the dose-response equation. However, the steepness of dose-response slope of the anesthetic and its Hill coefficient have not been linked to a brain network model that can explain anesthetic effects at the network level. In the analytic study, we proposed for the first time the relationship between the dose-response slope of the anesthetic, drug concentration, and coupling strength of the brain network. Two assumptions were made to link a Hill-type equation and a brain network model. The first assumption was that the anesthetic reduces coupling strengths between brain regions (in S1 Text equation S2). Empirical evidence supports this assumption with disintegrated brain functions at the network level under general anesthesia[22]. The second assumption was that drug concentration is inversely proportional to the brain network synchronization (in S1 Text equation S4) (that is, deep anesthesia induces an incoherent brain network). If these two assumptions are satisfied, then the dose-response slope of the anesthetic, drug concentration, and coupling strength of the brain network are related (in S1 Text equation S5). Interestingly, the effective coupling strength under anesthesia, *S*_*eff*_, is analytically equivalent to the adaptive feedback term, *SR*^*z*^, that has been used in the previous network models to facilitate explosive synchronization. Thus, in our brain network model under general anesthesia, the power of the feedback term, *z*, represents the anesthetic effect on the brain network (in S1 Text equation S11).

### Limitations

This study has several limitations. First, we did not perform a slow up- and down-titration in the ketamine experiment. As such, there was pharmacokinetic asymmetry that is independent of neurobiology or principles of hysteresis. However, the results at the network level during ketamine state transitions were similar to that of sevoflurane, which was up- and down-titrated, as well as the model. Second, we acknowledge that consciousness and unconsciousness cannot be trivially reduced to, respectively, synchronized and incoherent networks. It is temporal coordination rather than synchrony, per se, that is critical for consciousness. However, it is well known that functional brain networks become more modular during general anesthesia, as coordinated and synchronized interactions across the cortex break down. Thus, for the purposes of large-scale modeling, we consider it reasonable to assume that the conscious brain will have, in aggregate, more synchronized interactions compared to the unconscious brain. Third, our model focused on the coarse-grained features of brain network state transitions to identify the fundamental factors that produce and modulate hysteresis patterns. State transitions induced by general anesthetics are complex and the relationship between anesthetic concentration and behavioral response is nonlinear [54,55]. More realistic neural population models may extend the interpretation of our study to such nonlinear relationships. Fourth, there is still a large gap between the adaptive feedback term in the brain network model and the Hill coefficient in the dose-response curve of the anesthetic. However, precisely defining this relationship was beyond the scope of the current study.

### Conclusion

We characterized hysteresis phenomena in functional brain networks during anesthetic-induced state transitions. Our brain network model and its analytic derivation suggest that the asymmetry of synchronization suppression is the key mechanism of the hysteresis observed during loss and recovery of consciousness. Furthermore, we propose variance of frequencies and strength of the adaptive feedback process as the control parameters for modulating the onsets of state transitions in the human brain.

## Supporting information

S1 Text The analytic derivations and the descriptions about S1 Fig, S1 Table, and S2 Table. We analytically derived the relationship between anesthetic pharmacodynamics and the oscillator models in section 1, and also derived the extended oscillator model for the network hysteresis in section 2 and 3. The descriptions of figure and tables are in section 4 and 5.
(PDF)

S1 Fig The network thresholding and the number of isolated nodes. We tested the number of isolated nodes with increase of threshold in the construction of binary network. The threshold (0.1) was chosen to avoid isolated nodes in the EEG network in the baseline states (red dotted line).
(TIF)

S1 Table The significance levels of trajectory areas among frequency bands in ketamine experiment. The one-way ANOVA was applied and Tukey-Kramer correction was used for the multiple comparisons. (*P < 0.05, **P < 0.01, and ***P < 0.001).
(XLSX)

S2 Table The significance levels of trajectory areas among frequency bands in sevoflurane experiment. The one-way ANOVA was applied and Tukey-Kramer correction was used for the multiple comparisons. (*P < 0.05, **P < 0.01, and ***P < 0.001).
(XLSX)
