# Supplementary material for: Mechanisms of hysteresis in human brain networks during transitions of consciousness and unconsciousness: Theoretical principles and empirical evidence
Source: PLoS Comput Biol. 2018 Aug 30;14(8):e1006424. doi: 10.1371/journal.pcbi.1006424 (PMC6135517; doi:10.1371/journal.pcbi.1006424)
Supplement: S1 Text — We analytically derived the relationship between anesthetic pharmacodynamics and the oscillator models in section 1, and also derived the extended oscillator model for the network hysteresis in section 2 and 3. The descriptions of figure and tables are in section 4 and 5. (PDF) [file pcbi.1006424.s001.pdf]

Mechanisms of Hysteresis in Human Brain Networks during Transitions of Consciousness and Unconsciousness: Theoretical Principles and Empirical Evidence

Supplementary Text

Contents

1 Relationship between anesthetic pharmacodynamics and the oscillator models 1

2 Reducing the Stuart-Landau model to a Kuramoto model 2

3 Hysteresis from the Kuramoto model in complex networks 5

4 Selection of threshold in the construction of the connectivity network 8

5 Significance levels of trajectory areas among frequency bands 9

In this supplement, we first show the relationship between pharmacodynamic equations of anesthetics and the coupled oscillator models. We then show how the Stuart-Landau model can be reduced to the Kuramoto model as well as the mathematical analysis for the hysteresis in the phase transition of the Kuramoto model. Finally, we show the selection range of threshold to construct binary networks of functional connectivity and significance levels of trajectory areas among frequency bands in the empirical analysis.

1 Relationship between anesthetic pharmacodynamics and the oscillator models

In this section, we describe the relationship between pharmacodynamic equations for anesthetic effect and oscillator models. In the literature, anesthetic effects are usually described by the following Hill-type equations [1, 2]:

$$E_a \sim \frac{Ce^{\gamma}}{Ce_{50}^{\gamma} + Ce^{\gamma}}. \tag{S1}$$

Here, E_a denotes the effect of the given anesthetic, Ce is the concentration of the drug, and γ is a parameter determining the steepness of E_a, with respect to the changes of Ce. If a certain anesthetic drug has relatively higher γ compared to other drugs, that drug will have a steeper increasing function for E_a with respect to the increase of Ce.

In order to link Eq. (S1) with oscillator models, we make two independent assumptions. First, we assume that global connection strength between different areas of the brain is impeded by anesthetics in the following form:

$$S_{eff} = S(1 - E_a), \tag{S2}$$

where S_{eff} is the effective coupling strength, S is the baseline coupling strength without the anesthetics, and E_a is the effect of the given anesthetic defined as Eq. (S1). With this assumption, we proceed with the following:

$$
\begin{aligned}
S_{eff} =& S(1-E_a) \\
=& S\left[1-\frac{const.Ce^{\gamma}}{Ce_{50}^{\gamma}+Ce^{\gamma}}\right] = S\left[\frac{Ce_{50}^{\gamma}+(1-const.)Ce^{\gamma}}{Ce_{50}^{\gamma}+Ce^{\gamma}}\right] \\
=& S\left(\frac{Ce_{50}}{Ce}\right)^{\gamma}\frac{1+(1-const.)(Ce_{50})^{\gamma}}{1+(Ce_{50}/Ce)^{\gamma}} \sim \left(\frac{S}{Ce^{\gamma}}\right)\frac{1}{1+(1/Ce)^{\gamma}} \sim \frac{S}{Ce^{\gamma}}
\end{aligned}
\tag{S3}
$$

At this stage, we apply our second assumption, namely, that the effect of the drug concentration on the connection between different areas of the brain may be represented as the decrease of the synchronization between areas. The second assumption is derived from the synchronization changes during state transitions of empirical analysis in the alpha band of ketamine and sevoflurane experiments. Therefore, we assume that degree of the synchrony in the brain is inversely proportional to the drug concentration: the higher the concentration is, the lower the synchrony will be:

$$
Ce \sim \frac{1}{\tilde{R}}. \tag{S4}
$$

Here, $\tilde{R}$ represents an arbitrary measure of the synchrony. We will discuss the detailed form of the synchrony $\tilde{R}$ in the following section. We finally arrive at the following result:

$$
S_{eff} \sim \frac{S}{Ce^{\gamma}} \sim S\tilde{R}^{z}, \tag{S5}
$$

where $z \sim \gamma$.

2 Reducing the Stuart-Landau model to a Kuramoto model

In this section we describe the Stuart-Landau model implemented in complex networks and reduce the model to the Kuramoto model.

Stuart-Landau model is a canonical oscillator model for a large class of oscillators, i.e., the ones that exhibit Hopf bifurcation [3]. It is written as:

$$\dot{z}_j = \{\lambda_j + i\omega_j - (\sigma_j + i\gamma_j)|z_j|^2\}z_j + S\sum_{k=1}^{N} A_{jk}z_k, \qquad k = 1, 2, ..., N, \quad \text{(S6)}$$

where complex variable $\dot{z}_j(t)$ describes the state of jth oscillator.

In our analysis, we assume that λ, ω, σ, and γ are all nonnegative. Then the equation yields stable limit cycle from the supercritical Hopf bifurcation [4, 5]. A stable limit cycle appears via superciritical Hopf bifurcation when $\lambda_j > 0$. Dynamics of the equations for $\gamma = 0$ and $\gamma \neq 0$ are topologically equivalent, so the value of γ is often irrelevant. For ease of analysis, we set $\gamma = 0$ and $\sigma = 1$. Also, we add time-delay τ_{jk} between nodes j and k, which plays a crucial role in a network of neural mass. For each node j, the dynamics will be:

$$\dot{z}_j(t) = \{\lambda_j + i\omega_j - |z_j(t)|^2\}z(t) + S\sum_{k=1}^{N} A_{jk}z_k(t - \tau_{jk}), \quad \text{(S7)}$$

at time t. Eq. (S7) can be separated into two variables:

$$\dot{r}_j = \{\lambda_j - |z_j|^2\}r_j + S\sum_{k=1}^{N} A_{jk}r_k \cos(\theta_k(t - \tau_{jk}) - \theta_j(t)), \quad \text{(S8)}$$

$$\dot{\theta}_j = \omega_j + S\sum_{k=1}^{N} A_{jk}\frac{r_k}{r_j} \sin(\theta_k(t - \tau_{jk}) - \theta_j(t)). \quad \text{(S9)}$$

$r_j(t)$ is the amplitude of node j, and $\theta_j(t)$ is the phase of node j at time t. As discussed in the main paper, we add a so-called *feedback* term to the Eq. (S9) [6], to simulate the effect of anesthetics perturbing the connection between neural masses:

$$\dot{r}_j = \{\lambda_j - |z_j|^2\}r_j + S\sum_{k=1}^{N} A_{jk}r_k \cos(\theta_k(t - \tau_{jk}) - \theta_j(t)), \quad \text{(S10)}$$

$$\dot{\theta}_j = \omega_j + R_j^z S\sum_{k=1}^{N} A_{jk}\frac{r_k}{r_j} \sin(\theta_k(t - \tau_{jk}) - \theta_j(t)). \quad \text{(S11)}$$

Here, R_j^z is the feedback term, defined as:

$$R_j^z = \left| R_j e^{i\tilde{\Theta}_j} \right|^z \equiv \left| \frac{1}{2} \left(e^{i\theta_j} + \frac{1}{N} \sum_{k=1}^{N} e^{i\theta_k} \right) \right|^z . \quad \text{(S12)}$$

Here, z will be a positive number. We call R_j a *node synchrony* of j. The node synchrony will measure how much the phase of node j is synchronized with other nodes, yielding a value of 1 if the node j is in total synchrony with other nodes (*i.e.*, having a same value of phase), and approaching 0 when the distribution of its phase and other nodes' phases are close to random.

By adding the node synchrony R_j^z as the feedback term, we are assuming that the $\tilde{R}$ from Eq. (S5) can be well represented by such a term. We choose node synchrony R_j^z as the representative for $\tilde{R}$ based on the similarity of the model simulation results compared with the experimental analysis as shown in the main manuscript. It is possible that other feedback terms may also resemble similarity with the experimental analysis.

When we assume weak coupling in the vicinity of critical coupling strength [3], the amplitude of the oscillators become similar to each other. Taking the assumption that $r_j(t) = r_k(t)$ for $k = 1, 2, ..., N$, Eq. (S10) and Eq. (S11) are reduced to:

$$\dot{\theta}_j = \omega_j + R_j^z S \sum_{k=1}^{N} A_{jk} \sin(\theta_k(t-\tau) - \theta_j(t)), \qquad j = 1, 2, ..., N, \quad \text{(S13)}$$

which is the Kuramoto model with the feedback term. We make a further approximation of this equation: if the time delays between the oscillators τ_{jk} are similar or smaller in their order of magnitude compared to their oscillatory period, the effect of the delay can be represented by a phase delay term rather than explicit time delay term [7]. Assuming that the phase delay between oscillators are of same magnitude β, we arrrive at the following equation:

$$\dot{\theta}_j = \omega_j + R_j^z S \sum_{k=1}^{N} A_{jk} \sin(\theta_k - \theta_j - \beta), \qquad j = 1, 2, ..., N. \quad \text{(S14)}$$

3 Hysteresis from the Kuramoto model in complex networks

The Kuramoto model is the canonical model for all oscillators and widely used in the literature as an approximation for more complex and realistic systems [8, 9]. We first introduce local order parameter r_i:

$$r_j e^{i\Theta_j} = \frac{1}{k_j} \sum_{k=1}^{N} A_{jk} e^{i\theta_k}, \tag{S15}$$

and also global order parameter R:

$$Re^{i\Psi} = \frac{1}{N} \sum_{j=1}^{N} e^{i\theta_j}, \tag{S16}$$

where k_i is the sum of coupling to the oscillator j defined as $k_i = \sum_{j=1}^{N} A_{ij}$. Global order parameter R is a measure of synchrony for the entire system and will yield values 1 in the case of perfect synchrony among the nodes' phases, and 0 in case of a complete asynchrony. Local order parameter r_i of the oscillator i is a measure of synchrony among the oscillators connected to the oscillator i. The value will be 1 if they are in perfect synchrony and 0 if they are incoherent.

Using the local order parameter r_i, we can rewrite Eq. (S14) to the following form:

$$\dot{\theta}_j = \omega_j + R_j^z S k_j r_j \sin(\Theta_j - \theta_j - \beta), \qquad j = 1, 2, ..., N. \tag{S17}$$

In the analysis of the Eq. (S17), we follow mean-field approximation methods used in such references as [10, 11, 12]. Similar analysis was done for the Kuramoto model with the feedback terms in references [6] and [13]. Substituting Ψ from Eq. (S16) into Eq. (S17), yields

$$\dot{\theta}_j = \omega_j + R_j^z S k_j r_j \sin(\Psi - \theta_j - \beta), \tag{S18}$$

Let Ω denote the frequency of the population oscillation of Eq. (S18) after the system approaches a stationary state and let $\phi_j = \theta_j - \Omega t$ represent the phase of oscillator j relative to the average oscillation. The Eq. (S18) can then be rewritten using the order parameter defined in Eq. (S16) as follows:

$$\dot{\phi}_j = \omega_j - \Omega + R_j^z S k_j r_j \sin(\Phi - \phi_j - \beta), \qquad j = 1, 2, ..., N, \tag{S19}$$

where $\Phi = \Psi - \Omega t$. The mean-field method approximates r_j with R:

$$\dot{\phi}_j = \Delta_j + R_j^z S k_j R \sin(\Phi - \phi_j - \beta), \tag{S20}$$

where $\Delta_j = \omega_j - \Omega$.

The system exhibits a *partially locked state*. As studied in previous works [10, 11], for a non-zero coupling strength S there exist two groups of oscillators with different behaviors. Oscillators in the *phase-locked group* have the same frequency and thus their phase differences remain constant at each time point. Oscillators in *drifting group* cannot have same frequencies with the first group and thus do not phase-lock. They drift with different phase differences from one time point to another. The oscillators that can satisfy $\dot{\phi}_j = 0$ will be phase-locked: *i.e.*, the amplitude of the coupling terms must be equal or larger than that of the inherent terms:

$$R_j^z S k_j R \geq |\Delta_j|. \tag{S21}$$

The oscillators satisfying this condition will asymptotically approach a stable solution obtained from the following equation:

$$\omega_j - \Omega = R_j^z S k_j R \sin(\phi_j - \Phi + \beta). \tag{S22}$$

We can rearrange this equation to the following form:

$$\phi_j = \sin^{-1}\left(\frac{\Delta_j}{R_j^z S k_j R}\right) + \Phi - \beta, \tag{S23}$$

where $\Delta_j = \omega_j - \Omega$. Oscillators with $R_j^z S k_j R < |\Delta_j|$ cannot satisfy the condition $\dot{\phi}_j = 0$. They are the oscillators in the drifting group.

We will assume a symmetric probability distribution for the inherent frequencies of the oscillators ω_j. Also assuming that the drifting oscillators do not contribute to the order parameter R (an assumption based on previous studies [6, 13]), we can calculate the order parameter R from Eq. (S23). First, from Eq. (S16), we note the following:

$$Re^{i\Phi} e^{i\beta} = \frac{1}{N\langle k \rangle} \sum_{j=1}^{N} k_j e^{i\phi_j} e^{i\beta}, \tag{S24}$$

where $\langle k \rangle$ denotes the average degree of the oscillators. Taking the real component of Eq. (S24) and contributions from the phase-locked population only, we arrive:

$$R\cos(\beta) = \frac{1}{N\langle k\rangle} \sum_{|\Delta_j| \le R_j^z S k_j R} k_j \cos(\phi_j - \Phi + \beta). \tag{S25}$$

Substituting Eq. ((S24)) into Eq. ((S25)), we arrive at the following:

$$R = \frac{1}{N\langle k\rangle \cos\beta} \sum_{|\Delta_j| \le R_j^z S k_j R} k_j \sqrt{1 - \left(\frac{\Delta_j}{R_j^z S k_j R}\right)^2}. \tag{S26}$$

In the thermodynamic limit ($N \to \infty$), we replace the summation by the integration:

$$R = \frac{1}{\langle k\rangle \cos\beta} \int_{|\Delta| \le R_j^z S k R} g(k) h(\Delta) k \sqrt{1 - \left(\frac{\Delta}{R_j^z S k R}\right)^2} d\Delta dk, \tag{S27}$$

where $g(k)$ is the probability distribution of the degree of the nodes and $h(\Delta)$ the probability distribution of initial frequencies (minus Ω).

We take one final step in our analysis. Inspecting the definition of R_j^z Eq. (S12), we can rewrite as the following:

$$R_j^z = \left|\frac{1}{2}\left(e^{i\theta_j} + Re^{i\Psi}\right)\right|^z. \tag{S28}$$

For oscillators locked near the phase Ψ (such as the phase-locked group oscillators with sufficiently strong coupling strength S), R_j^z can be better approximated by R compared to r_j. Therefore, the range of the integral in Eq. (S28) can be written as the following in good approximation:

$$|\Delta| \le SkR^{z+1}. \tag{S29}$$

This integral range may be a good approximation for a range of feedback terms if the terms can be well approximated by the mean-field method.

Our model of coupled oscillators with a feedback term exhibits a hysteresis in the value of R when we control S: there exists path dependency and therefore the curves are disparate when S is increased and decreased. With Eq. (S27), we can explain such hysteresis in comparison to a model with no feedback term.

We follow the argument reported in Ref. [13] in comparing the above model and regular Kuramoto model without the feedback term:

$$\dot{\theta}_j = \omega_j + S\sum_{k=1}^{N} A_{jk}\sin(\theta_k - \theta_j - \beta) \qquad j = 1, 2, ..., N. \tag{S30}$$

With mean-field approach, we obtain:

$$R = \frac{1}{\langle k\rangle \cos\beta}\int_{|\Delta|\le SkR} g(k)h(\Delta)k\sqrt{1-\left(\frac{\Delta}{SkR}\right)^2}\, d\Delta dk. \tag{S31}$$

We note that in the case of regular Kuramoto models, we do not observe hysteresis in the transition of R as we increase/decrease k: the value of R is uniquely defined against a value of k.

Comparing this result with the one having a feedback term Eq. (S27), we note that the main difference lies in the range of integral: $|\Delta| \le SkR^{z+1}$ vs. $|\Delta| \le SkR$. When we decrease S from a sufficiently large value, the system are already in a highly synchronous state, regardless of the existence of the feedback term: $R^z \approx R \approx 1$. However, when we increase S from an incoherent state ($R \gtrapprox 0$), the difference between R^z and R is magnified for R^z is significantly smaller than R. Therefore, the integral range in two systems (Eq. (S27)) and Eq. (S31)) will significantly differ, meaning that the number of the oscillators of the system belonging to the phase-locked group will differ too: in the feedback system Eq. (S27), the formation of larger synchronized clusters are delayed until we increase coupling strength S to a higher value. The synchronized cluster will be formed abruptly until it is unavoidable. Such asymmetry of the integral range compared to the regular Kuramoto model predicts path-dependency/hysteresis as confirmed by our simulations.

4 Selection of threshold in the construction of the connectivity network

To remove spurious connections derived from EEG analysis, we subtract the median connectivity of 20 surrogate data sets from original connectivity and apply the threshold 0.1 to construct a binary network. We choose the threshold value as 0.1 to avoid isolated nodes in the EEG network in the baseline states. S1 Fig shows the increase of isolated nodes with increased values of threshold in the resting state of the ketamine experiment.

5 Significance levels of trajectory areas among frequency bands

The significance levels of trajectory areas among frequency bands were calculated with a one-way ANOVA and Tukey-Kramer correction for multiple comparisons, and are shown respectively for ketamine and sevoflurane data in S1 Table and S2 Table. The numbers in the table represent the median frequency of each frequency band, and the width of each frequency band is 2 Hz. The results show that the trajectory areas are significantly larger in the range of alpha band than other frequency bands.

Supporting References

[1] Louizos, C., Yáñez, J. A., Forrest, M. L. & Davies, N. M. Understanding the hysteresis loop conundrum in pharmacokinetic/pharmacodynamic relationships. *J. Pharm. Pharm. Sci.* **17**, 34–91 (2014).

[2] Pleuvry, B. J. Hysteresis in drug response. Anaesth. *Intensive Care Med.* **6**, 286–287 (2005).

[3] Hoppensteadt, F. C. & Izhikevich, E. M. *Weakly Connected Neural Networks.* (Springer-Verlag, 1997).

[4] Selivanov, A.A., Lehnert J., Dahms T., Hövel P., Fradkov A. L. & Sch öll E. Adaptive synchronization in delay-coupled networks of Stuart-Landau oscillators. *Phys. Rev. E.* **85**, 016201 (2012).

[5] Bergner A., Frasca M., Sciuto G., Buscarino A., Ngamga E. J., Fortuna L. & Kurths J. Remote synchronization in star networks. *Phys. Rev. E.* **85**, 026208 (2012).

[6] Filatrella, G., Pedersen, N. F. & Wiesenfeld, K. Generalized coupling in the Kuramoto model. *Phys. Rev. E.* **75**, 17201 (2007).

[7] Izhikevich, E. M. Phase models with explicit time delays. *Phys Rev E.* **58**, 905-908 (1998).

[8] Kuramoto, Y. *International Symposium on Mathematical Problems in Theoretical Physics, Lecture Notes in Physics, 39.* (Arakai, H.), 420-422 (Springer-Verlag, 1975).

[9] Strogatz, S. H. *Nonlinear Dynamics and Chaos. 1st ed.* (Westview Press, 1994).

[10] Ko, T.-W. & Ermentrout, G. B. Partially locked states in coupled oscillators due to inhomogeneous coupling. *Phys Rev E.* **78**, 016203 (2008).

[11] Moon, J.-Y., Lee, U., Blain-Moraes, S. & Mashour, G. A. General relationship of global topology, local dynamics, and directionality in large-scale brain networks. *PLoS Comput Biol.* **11**, e1004225 (2015).

[12] Moon, J.-Y. et al. Structure shapes dynamics and directionality in diverse brain networks: Mathematical principles and empirical confirmation in three species. *Sci. Rep.* **7**, 1–11 (2017).

[13] Zhang, X., Boccaletti, S., Guan, S. & Liu, Z. Explosive synchronization in adaptive and multilayer networks. *Phys. Rev. Lett.* **114**, 38701 (2015).
