# Supplementary figures and images for: Mechanisms of hysteresis in human brain networks during transitions of consciousness and unconsciousness: Theoretical principles and empirical evidence
Source: PLoS Comput Biol. 2018 Aug 30;14(8):e1006424. doi: 10.1371/journal.pcbi.1006424 (PMC6135517; doi:10.1371/journal.pcbi.1006424)

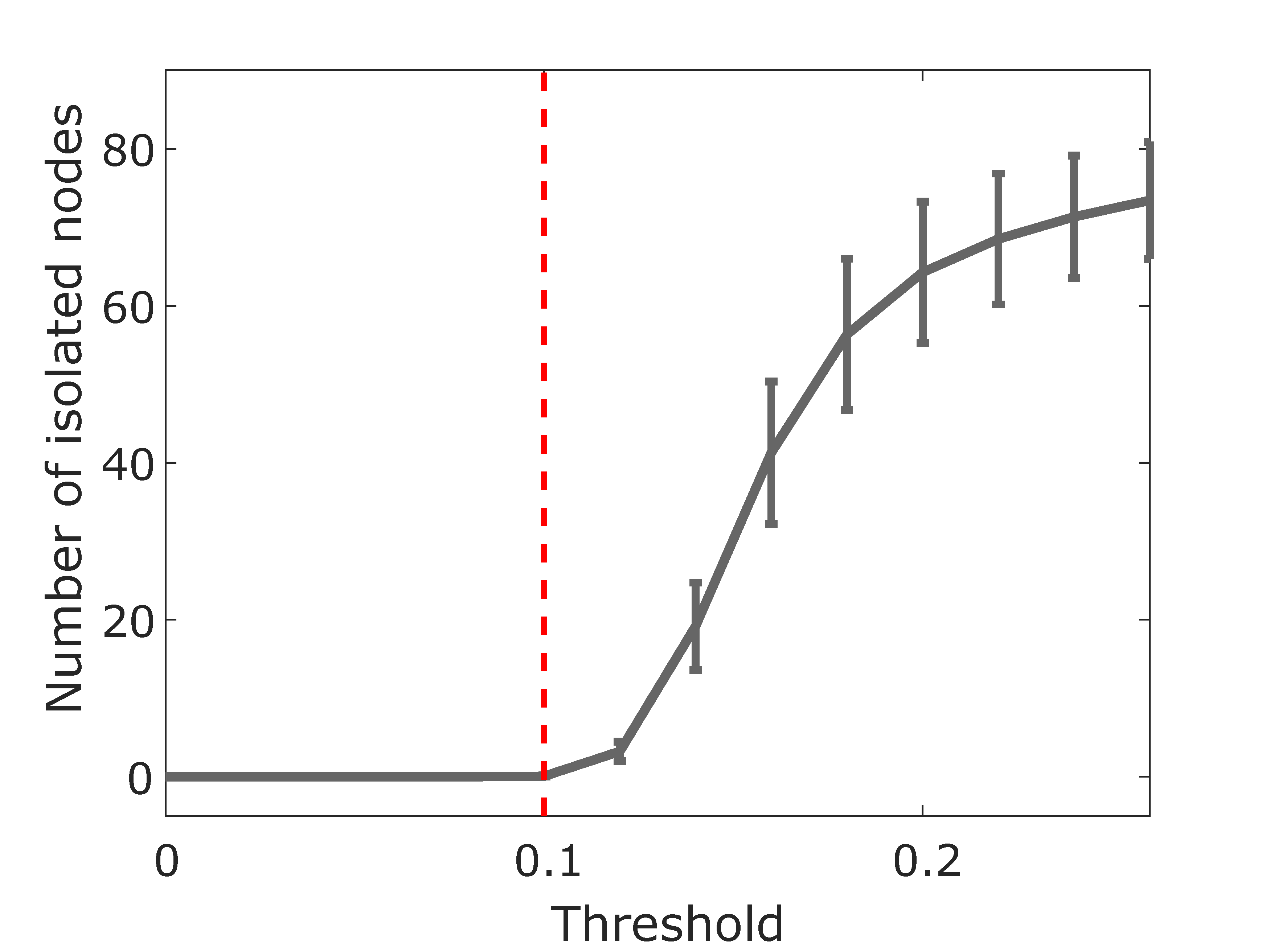

Supplement: S1 Fig — We tested the number of isolated nodes with increase of threshold in the construction of binary network. The threshold (0.1) was chosen to avoid isolated nodes in the EEG network in the baseline states (red dotted line). (TIF) [file pcbi.1006424.s002.tif]
